# *Porphyromonas gingivalis*, *Aggregatibacter actinomycetemcomitans*, and *Treponema denticola / Prevotella intermedia* Co-Infection Are Associated with Severe Periodontitis in a Thai Population

**DOI:** 10.1371/journal.pone.0136646

**Published:** 2015-08-27

**Authors:** Kitti Torrungruang, Supawadee Jitpakdeebordin, Orawan Charatkulangkun, Yingampa Gleebbua

**Affiliations:** 1 Department of Microbiology, Faculty of Dentistry, Chulalongkorn University, Bangkok, Thailand; 2 Laboratory Animal Facility, Faculty of Science, Mahidol University, Bangkok, Thailand; 3 Department of Periodontology, Faculty of Dentistry, Chulalongkorn University, Bangkok, Thailand; 4 Health Division, Medical and Health Department, Electricity Generating Authority of Thailand, Nonthaburi, Thailand; University of Toronto, CANADA

## Abstract

Periodontitis is a polymicrobial infection of tooth-supporting tissues. This cross-sectional study aimed to examine the associations between five target species and severe periodontitis in a Thai population. Using the CDC/AAP case definition, individuals diagnosed with no/mild and severe periodontitis were included. Quantitative analyses of *Aggregatibacter actinomycetemcomitans* (*Aa*), *Porphyromonas gingivalis* (*Pg*), *Tannerella forsythia* (*Tf*), *Treponema denticola* (*Td*), and *Prevotella intermedia* (*Pi*) in subgingival plaque were performed using real-time polymerase chain reaction. The association between target species and severe periodontitis was examined using logistic regression analysis. The study subjects comprised 479 individuals with no/mild periodontitis and 883 with severe periodontitis. Bacterial prevalence and quantity were higher in subjects with severe periodontitis than in those with no/mild disease. In the fully adjusted model, all species except *Tf* showed a dose-dependent relationship with periodontitis. The mere presence of *Pg*, even in low amount, was significantly associated with severe periodontitis, while the amount of *Aa*, *Td*, and *Pi* had to reach the critical thresholds to be significantly associated with disease. Compared to individuals with low levels of both *Td* and *Pi*, high colonization by either *Td* or *Pi* alone significantly increased the odds of having severe periodontitis by 2.5 (95%CI 1.7–3.5) folds. The odds ratio was further increased to 14.8 (95%CI 9.2–23.8) in individuals who were highly colonized by both species. Moreover, the presence of *Pg* and high colonization by *Aa* were independently associated with severe periodontitis with odds ratios of 5.6 (95%CI 3.4–9.1) and 2.2 (95%CI 1.5–3.3), respectively. Our findings suggest that the presence of *Pg* and high colonization by *Aa*, *Td*, and *Pi* play an important role in severe periodontitis in this study population. We also demonstrate for the first time that individuals co-infected with *Td* and *Pi* were more likely to have periodontitis than were those infected with a single pathogen.

## Introduction

Periodontitis is an inflammatory disease of tooth-supporting tissues, characterized by loss of connective tissue attachment and alveolar bone. The primary etiologic agent of periodontitis is subgingival plaque bacteria. It is generally accepted that periodontitis is a polymicrobial disease, where complex interactions between specific pathogens are more relevant to disease development than are individual species [1,2]. Using cluster analysis and community ordination techniques, Socransky et al. identified five microbial complexes, which are repeatedly found together in subgingival biofilm [1]. Among these, the “red complex”, consisting of *Porphyromonas gingivalis*, *Tannerella forsythia*, and *Treponema denticola*, is considered the most pathogenic microbial complex. Several studies across different populations have demonstrated that the presence and amount of these species or their combinations are associated with disease parameters, including probing depth, bleeding on probing, attachment loss, and bone loss [1,3–7].

In addition to the red complex, different combinations of bacterial species have been reported to be important for periodontitis. The salivary presence of *Aggregatibacter actinomycetemcomitans* with *P*. *gingivalis* and *T*. *denticola* has been shown to contribute to deepened pockets in a Finnish population [8]. Another Finnish study reported that a combination between *A*. *actinomycetemcomitans*, *P*. *gingivalis*, and *Prevotella intermedia* showed the strongest association with disease [9]. Furthermore, the presence of *Porphyromonas endodontalis* / *Porphyromonas* spp. and *T*. *forsythia*, and the absence of *Prevotella denticola* and *Neisseria polysaccharea* have been identified as risk indicators of periodontal disease in Brazilians [2]. Despite the methodological differences between the studies, these findings suggest that a bacterial consortium involved in the development of periodontitis may vary in different populations. Therefore, it is important to identify bacterial species associated with disease in a particular population in order to establish the preventive and therapeutic strategies suitable for this population.

It is believed that the irreversible destruction of periodontal tissues only occurs when bacterial levels exceed a critical threshold [10]. However, most microbial detection methods such as checkerboard DNA-DNA hybridization, immunological assays, and conventional end-point polymerase chain reaction (PCR) only provide qualitative analysis or at best relative quantification of target species. Real-time PCR provides advantages over these methods because it can determine not only the presence or absence, but also the absolute amount of microorganisms with a high sensitivity and a broad detection range [11]. Therefore, it can be used to identify the threshold required for each species to cause periodontal breakdown [12]. The aim of this study was to use real-time PCR for detection and quantification of five periodontal pathogens, i.e. *A*. *actinomycetemcomitans*, *P*. *gingivalis*, *T*. *forsythia*, *T*. *denticola*, and *P*. *intermedia* in a Thai population. Logistic regression analysis was used to determine the associations between the levels of these species and severe periodontitis, taking into account the effect of known confounding factors. The threshold effect of each species was examined, and the interaction between pathogenic species with regard to the odds of having severe periodontitis was investigated.

## Materials and Methods

### Study participants

This cross-sectional study is part of a cohort study conducted to identify risk factors for several systemic diseases among the employees of the Electricity Generating Authority of Thailand (EGAT) [13]. The subjects included individuals who work at EGAT headquarters in the Bangkok metropolitan area, and at three hydroelectric plants in northern and western Thailand. They were enrolled in the study from June to November 2003. The participants had at least six teeth, and did not require antibiotic prophylaxis for periodontal examinations. The study protocol was approved by the Ethics Committees of the Ramathibodi Hospital Faculty of Medicine at Mahidol University, and the Faculty of Dentistry at Chulalongkorn University. Written informed consent was obtained from each participant.

### Periodontal examinations

Periodontal examinations and sample collection were carried out as previously described [14]. Probing depth (PD) and gingival recession were measured at six sites per tooth (mesio-buccal, mid-buccal, disto-buccal, mesio-lingual, mid-lingual, and disto-lingual) in all fully erupted teeth except third molars and retained roots. Clinical attachment level (CAL) was calculated as the sum of PD and gingival recession. Subgingival plaque samples were collected using a sterile curette from mesio-buccal aspects of teeth in the right quadrants and from mesio-lingual aspects of teeth in the left quadrants. The samples from each subject were pooled and stored in 1 ml of sterile phosphate-buffered saline containing 0.01% thimerosal, and kept at -80°C until use.

### Real-time PCR

Bacterial genomic DNA was extracted from 200 μl of each sample using the QIAamp DNA Mini Kit (QIAGEN, Valencia, CA, USA). Bacterial quantification was carried out using 16S rRNA gene-based real-time PCR. Species-specific primers and probes for *A*. *actinomycetemcomitans* [11], *P*. *gingivalis* [15], *T*. *forsythia* [16], *T*. *denticola*, and *P*. *intermedia* [17] were used as previously described. PCR assay was performed in a 20-μl final volume, containing 10 μl of LightCycler 480 Probes Master (Roche Diagnostics, Mannheim, Germany), forward and reverse primers at 0.25 μM each, 0.25 μM Taqman probe, and 5 μl of bacterial DNA sample. The cycling protocol included an enzyme activation step at 95°C for 10 min, followed by 40 cycles of amplification, 95°C for 15 sec and 60°C for 1 min. 

### Standard curve for bacterial quantification

PCR quantification standards were prepared using plasmids containing the amplified rRNA region of each target bacterium. The reference strains used for preparing the standards were *A*. *actinomycetemcomitans* ATCC29522, *P*. *gingivalis* ATCC33277, *T*. *forsythia* ATCC43037, *T*. *denticola* ATCC35405, and *P*. *intermedia* ATCC25611. PCR amplicons obtained from these species were cloned into separate plasmid vectors using the TOPO TA Cloning Kit (Invitrogen Corp. Carlsbad, CA, USA). Insertion was confirmed by restriction enzyme analysis and agarose gel electrophoresis.

The plasmid standards were serially diluted from 10^10^ to 10 DNA copies, and amplified using the protocol described above. The standard curve was generated as a plot between the cycle number at the crossing point (Cp) and the initial plasmid DNA copies. PCR efficiency was greater than 95%, and errors from tube-to-tube variation were less than 0.03. Using the standard curve, absolute quantity of each target species was calculated as DNA copies per reaction. The lower limit of detection was 10 DNA copies. Due to inter-species variations in the copy number of 16S rRNA genes, DNA copies were divided by rRNA gene copies per cell, i.e. 2 for *T*. *forsythia* and *T*. *denticola*, 4 for *P*. *gingivalis* and *P*. *intermedia*, and 6 for *A*. *actinomycetemcomitans* [18], and was then multiplied by 200 (sample dilution in the PCR assay) to obtain the total cell number of each species.

### Statistical analysis

Using the Centers for Disease Control and Prevention (CDC)/American Academy of Periodontology (AAP) case definition [19], subjects who were diagnosed with no/mild periodontitis and severe periodontitis were included for analysis. Descriptive data were expressed as frequencies and percentages for categorical variables, and as means and standard deviations for continuous variables. Bacterial quantity was reported as median number of bacterial cells and interquartile ranges. Smoking status was assessed by a self-reported questionnaire [20]. Diabetes mellitus was diagnosed based on fasting blood sugar of ≥126 mg/dl or taking anti-diabetic drugs during the past two weeks.

Differences between categorical variables were evaluated using chi-square tests. Differences between continuous variables were analyzed using t-tests or Mann-Whitney U tests as appropriate. The association between the levels of each target species was examined using Spearman’s rank correlation, in which the correlation coefficients (r) of 0.60 or higher were considered a strong association. Odds ratios (OR) and 95% confidence intervals (CI) were calculated by logistic regression analyses to examine the associations between target microorganisms and severe periodontitis. Each species was included in the analyses as a four-level or six-level categorical variable. Level 0 represented PCR-negative subjects, while the higher levels were categorized according to the tertile or quintile distribution of the number of bacterial cells in PCR-positive subjects. The critical threshold for each species was identified based on the lowest bacterial level that reached statistical significance. All regression analyses were adjusted for known confounders of periodontitis, including age, gender, education level, number of remaining teeth, smoking status, and diabetes. To determine the ability to discriminate between no/mild and severe periodontitis, negative and positive predictive values were calculated for the microorganisms included in the final model. Statistical analysis was performed using SPSS version 17.0 software (IBM, Chicago, IL, USA). P value <0.05 was considered statistically significant.

## Results

The study subjects comprised 479 individuals with no/mild periodontitis and 883 individuals with severe periodontitis (Table 1). Individuals who had severe periodontitis were significantly older, less educated, and had fewer remaining teeth than those with no/mild disease (P <0.001). In addition, the severe periodontitis group had significantly higher proportions of males, smokers, and diabetics (P <0.001).

**Table 1:** Demographic and clinical characteristics of study participants according to periodontal status.

Characteristics	No/mild periodontitis	Severe periodontitis
	N = 479	N = 883
Age (years; mean ± s.d)^a^	46.6 ± 4.4	49.3 ± 4.9
Gender, N (%)^b^		
Female	211 (44.1)	121 (13.7)
Male	268 (55.9)	762 (86.3)
Education, N (%)^b^		
<Bachelor degree	173 (36.1)	660 (74.7)
≥Bachelor degree	306 (63.9)	223 (25.3)
No. of remaining teeth (mean ± s.d)^a^	25.2 ± 3.3	23.1 ± 4.8
Smoking status, N (%)^b^		
Non-smokers	358 (74.7)	312 (35.3)
Former smokers	83 (17.3)	256 (29.0)
Current smokers	38 (7.9)	315 (35.7)
Diabetes, N (%)^b^		
No	462 (96.5)	781 (88.4)
Yes	17 (3.5)	102 (11.6)

^a^ Significant differences between groups at *P* <0.001, analyzed using t tests.

^b^ Significant differences between groups at *P* <0.001, analyzed using chi-square tests.

### Quantitative analysis of target species

Quantitative real-time PCR analyses of subgingival plaque samples are presented in Table 2. In subjects with no/mild periodontitis, *T*. *forsythia* was the most frequently detected bacteria (92%), followed by *T*. *denticola* (82%), *P*. *intermedia* (70%), *P*. *gingivalis* (62%), and *A*. *actinomycetemcomitans* (26%). In the severe periodontitis group, all species were found in the majority of the subjects (>95%), except for *A*. *actinomycetemcomitans*, which was harbored in half of the subjects. Among five species, the black-pigmented bacteria, *P*. *gingivalis* and *P*. *intermedia*, demonstrated the highest median levels in the PCR-positive subjects of both groups, whereas the lowest median level was found for *A*. *actinomycetemcomitans*. The prevalence and quantity of all five species were significantly greater in individuals with severe periodontitis than in those with no/mild disease (P <0.001).

**Table 2:** Prevalence of target species and their quantities in PCR-positive subjects.

Microorganisms	No/mild periodontitis	Severe periodontitis
	N = 479	N = 883
*Aa*		
Prevalence, N (%)^a^	125 (26.1)	444 (50.3)
Median bacterial cells	3.4 x10^4^	2.1 x10^5^
(interquartile range)^b^	(1.8 x10^3^–3.9 x10^5^)	(2.1 x10^4^–1.2 x10^6^)
*Pg*		
Prevalence, N (%)^a^	299 (62.4)	849 (96.1)
Median bacterial cells	7.0 x10^6^	6.3 x10^7^
(interquartile range)^b^	(5.2 x10^5^–3.8 x10^7^)	(1.8 x10^7^–1.7 x10^8^)
*Tf*		
Prevalence, N (%)^a^	441 (92.1)	870 (98.5)
Median bacterial cells	5.9 x10^6^	1.7 x10^7^
(interquartile range)^b^	(2.0 x10^6^–1.4 x10^7^)	(7.2 x10^6^–3.7 x10^7^)
*Td*		
Prevalence, N (%)^a^	391 (81.6)	864 (97.8)
Median bacterial cells	3.2 x10^6^	2.1 x10^7^
(interquartile range)^b^	(2.8 x10^5^–1.3 x10^7^)	(6.6 x10^6^–5.0 x10^7^)
*Pi*		
Prevalence, N (%)^a^	334 (69.7)	850 (96.3)
Median bacterial cells	9.4 x10^6^	5.3 x10^7^
(interquartile range)^b^	(7.6 x10^5^–4.0 x10^7^)	(1.7 x10^7^–1.4 x10^8^)

Abbreviations: *Aa* = *A*. *actinomycetemcomitans*, *Pg* = *P*. *gingivalis*, *Tf* = *T*. *forsythia*, *Td* = *T*. *denticola*, and *Pi* = *P*. *intermedia*.

^a^ Significant differences between groups at *P* <0.001, analyzed using chi-square tests.

^b^ Significant differences between groups at *P* <0.001, analyzed using Mann-Whitney U tests.

### Inter-species correlations

Correlations between the levels of each target species are presented in Table 3. Among subjects with no/mild periodontitis, strong correlations were found only between *T*. *forsythia* and *T*. *denticola*. Among subjects with severe periodontitis, strong correlations were observed between members of the red complex and between *P*. *intermedia* and the red complex species. 

**Table 3:** Inter-species correlations in subjects with no/mild periodontitis (below the diagonal) and severe periodontitis (above the diagonal).

	*Aa*		*Pg*		*Tf*		*Td*		*Pi*	
	r	P value	r	P value	r	P value	r	P value	r	P value
*Aa*			0.11	0.001	0.12	<0.001	0.12	<0.001	0.13	<0.001
*Pg*	0.10	0.036			**0.79**	<0.001	**0.78**	<0.001	**0.63**	<0.001
*Tf*	0.10	0.028	0.42	<0.001			**0.79**	<0.001	**0.67**	<0.001
*Td*	0.16	<0.001	0.46	<0.001	**0.66**	<0.001			**0.68**	<0.001
*Pi*	0.11	0.020	0.43	<0.001	0.39	<0.001	0.41	<0.001		

Abbreviations: *Aa* = *A*. *actinomycetemcomitans*, *Pg* = *P*. *gingivalis*, *Tf* = *T*. *forsythia*, *Td* = *T*. *denticola*, *Pi* = *P*. *intermedia*, and r = Spearman rank correlation coefficients.

Strong correlations are indicated in bold.

### Association between bacterial levels and periodontitis

The relationship between the levels of target species and severe periodontitis was analyzed using logistic regression models (Table 4). In partially adjusted models, each species was separately included in the analyses, adjusting for known confounders of disease. A strong association was observed between all five microorganisms and periodontitis (P <0.001). The odds of having disease increased as the bacterial levels increased. When these species were included in the model simultaneously, *T*. *forsythia* did not reach statistical significance (P >0.05), and thus was removed from the model. Among the remaining four species, *P*. *gingivalis* showed the strongest association with disease. Interestingly, the mere presence of *P*. *gingivalis*, even at its lowest level, significantly increased the odds of having severe periodontitis. For *A*. *actinomycetemcomitans*, *T*. *denticola*, and *P*. *intermedia*, their amount had to reach the critical thresholds to be significantly associated with disease. These thresholds were 3.3x10^4^, 1.9x10^7^, and 5.1x10^6^ cells, respectively. Individuals were considered as highly colonized by certain species if bacterial levels were at or above these thresholds.

**Table 4:** Association between levels of target species and severe periodontitis.

Bacterial levels^a^	No. of subjects	No. (%) with severe periodontitis	Partially adjusted OR (95% CI)^b^	P value^b^	Fully adjusted OR (95% CI)^c^	P value^c^
*Aa*						
0	793	439 (55.4)	1	-	1	-
1	190	128 (67.4)	1.5 (1.0–2.2)	0.063	1.5 (1.0–2.5)	0.070
2	190	153 (80.5)	2.7 (1.7–4.2)	<0.001	**1.7 (1.0–2.8)**	**0.045**
3	189	163 (86.2)	5.6 (3.5–9.1)	<0.001	3.1 (1.7–5.5)	<0.001
*Pg*						
0	214	34 (15.9)	1	-	1	-
1	229	96 (41.9)	2.8 (1.7–4.8)	<0.001	**3.2 (1.8–5.8)**	**<0.001**
2	230	158 (68.7)	8.6 (5.0–14.7)	<0.001	6.5 (3.6–11.9)	<0.001
3	231	183 (79.2)	12.8 (7.3–22.5)	<0.001	6.0 (3.2–11.4)	<0.001
4	229	198 (86.5)	24.3 (13.4–44.1)	<0.001	6.7 (3.4–13.3)	<0.001
5	229	214 (93.4)	61.0 (30.3–122.8)	<0.001	8.0 (3.4–18.8)	<0.001
*Tf*						
0	51	13 (25.5)	1	-		
1	262	105 (40.1)	1.3 (0.6–3.1)	0.478		
2	262	159 (60.7)	3.4 (1.5–7.7)	0.004		
3	264	172 (65.2)	3.8 (1.6–8.6)	0.002		
4	261	200 (76.6)	8.4 (3.6–19.5)	<0.001		
5	262	234 (89.3)	22.7 (9.4–54.8)	<0.001		
*Td*						
0	107	19 (17.8)	1	-	1	-
1	251	96 (38.2)	1.8 (1.0–3.5)	0.069	1.5 (0.7–3.0)	0.302
2	251	141 (56.2)	3.3 (1.7–6.3)	<0.001	1.4 (0.6–2.9)	0.424
3	251	177 (70.5)	7.2 (3.7–13.9)	<0.001	2.0 (0.9–4.2)	0.086
4	252	210 (83.3)	14.0 (7.1–27.7)	<0.001	**3.2 (1.4–7.2)**	**0.004**
5	250	240 (96.0)	74.5 (31.3–177.2)	<0.001	10.4 (3.6–30.1)	<0.001
*Pi*						
0	178	33 (18.5)	1	-	1	-
1	237	100 (42.2)	2.1 (1.2–3.6)	0.007	1.7 (0.9–3.2)	0.082
2	237	152 (64.1)	5.2 (3.0–8.9)	<0.001	**2.2 (1.2–4.2)**	**0.013**
3	238	191 (80.3)	10.9 (6.1–19.5)	<0.001	3.9 (2.0–7.6)	<0.001
4	237	192 (81.0)	14.9 (8.3–26.7)	<0.001	3.4 (1.7–6.8)	<0.001
5	235	215 (91.5)	37.0 (19.1–71.8)	<0.001	5.0 (2.3–11.2)	<0.001

Abbreviations: *Aa* = *A*. *actinomycetemcomitans*, *Pg* = *P*. *gingivalis*, *Tf* = *T*. *forsythia*, *Td* = *T*. *denticola*, and *Pi* = *P*. *intermedia*.

^a^ Bacterial level 0 represents PCR-negative subjects, while the higher levels are categorized according to the tertile or quintile distribution of the number of bacterial cells in PCR-positive subjects. The cut-off values are as follows: 3.3x10^4^ and 5.8x10^5^ for *Aa*; 4.4x10^6^, 2.6x10^7^, 6.7x10^7^, and 1.7x10^8^ for *Pg*; 3.1x10^6^, 8.7x10^6^, 1.7x10^7^, and 3.3x10^7^ for *Tf*; 1.7x10^6^, 7.6x10^6^, 1.9x10^7^, and 4.6x10^7^ for *Td*; and 5.1x10^6^, 2.3x10^7^, 5.6x10^7^, and 1.3x10^8^ for *Pi*.

^b^ Logistic regression analysis of each microorganism individually, adjusted for known confounders: age, gender, education, number of remaining teeth, smoking status, and diabetes.

^c^ Logistic regression analysis of multiple target species, adjusted for known confounders. The lowest bacterial level that reached statistical significance (P <0.05), is in boldface.

### Interaction between target species and periodontitis

To further examine the effect of inter-species interaction on periodontitis, each species was included in the logistic regression model as two-level categorical variables: “high” vs “low” colonization for *A*. *actinomycetemcomitans*, *T*. *denticola*, and *P*. *intermedia*, and “presence” vs “absence” for *P*. *gingivalis*. Interactions between these species were examined by introducing an interaction term into the model. The results showed that an interaction between *T*. *denticola* and *P*. *intermedia* was significantly associated with periodontitis (P = 0.005). Therefore, variables representing co-infection between these two species were included in the final model (Table 5). Compared to subjects who harbored low amount of both species, high colonization by either *T*. *denticola* or *P*. *intermedia* alone was significantly associated with severe periodontitis with OR of 2.5 (95% CI 1.7–3.5). The OR was further increased to 14.8 (95% CI 9.2–23.8) in individuals who were highly colonized by both species. Moreover, the presence of *P*. *gingivalis* and high colonization by *A*. *actinomycetemcomitans* were independently associated with periodontitis with ORs of 5.6 (95% CI 3.4–9.1) and 2.2 (95% CI 1.5–3.3), respectively.

**Table 5:** Logistic regression model of multiple species and their interaction with regard to the odds of having severe periodontitis.

Microorganisms	No. of subjects	No. (%) with severe periodontitis	Adjusted OR (95% CI)^a^	P value^a^
*Aa*				
Low level (reference)^b^	983	567 (57.7)	1	-
High level^c^	379	316 (83.4)	2.2 (1.5–3.3)	<0.001
*Pg*				
Absence (reference)	214	34 (15.9)	1	-
Presence	1148	849 (74.0)	5.6 (3.4–9.1)	<0.001
*Td* and *Pi*				
Both at low level (reference)^b^	386	121 (31.3)	1	-
High *Td* or *Pi* only^c^	503	324 (64.4)	2.5 (1.7–3.5)	<0.001
Both at high level^c^	473	438 (92.6)	14.8 (9.2–23.8)	<0.001

Abbreviations: *Aa* = *A*. *actinomycetemcomitans*, *Pg* = *P*. *gingivalis*, *Td* = *T*. *denticola*, and *Pi* = *P*. *intermedia*.

^a^ Logistic regression analysis, controlling for age, gender, education, number of remaining teeth, smoking status, diabetes, and the effect of other species.

^b^ “Low level” indicates the bacterial levels below these thresholds: 3.3x10^4^, 1.9x10^7^, and 5.1x10^6^ cells for *Aa*, *Td*, and *Pi*, respectively.

^c^ “High level” indicates the bacterial levels at or above the thresholds.

### Predictive values for health and disease

The predictive values for each species or their combination in discriminating between no/mild and severe periodontitis are shown in Table 6. The low negative predictive values were observed when *A*. *actinomycetemcomitans* or *T*. *denticola* were detected below the thresholds (42% and 50%, respectively). The value increased to 68% for low abundance of *P*. *intermedia* and 84% for the absence of *P*. *gingivalis*. The positive predictive value was relatively low for the presence of *P*. *gingivalis* (74%). The value increased to 79–90% for high colonization by *A*. *actinomycetemcomitans*, *T*. *denticola* or *P*. *intermedia*. The combination of all four species produced the highest negative and positive predictive values (91% and 97%, respectively).

**Table 6:** Predictive values of periodontal pathogens or their combination in discriminating between no/mild and severe periodontitis.

Microorganisms^a^	No/mild periodontitis	Severe periodontitis	NPV (%)	PPV (%)
Low *Aa*	416	567		
High *Aa*	63	316	42.3	83.4
*Pg* absent	180	34		
*Pg* present	299	849	84.1	74.0
Low *Td*	427	433		
High *Td*	52	450	49.7	89.6
Low *Pi*	282	133		
High *Pi*	197	750	68.0	79.2
*Pg* absent + low *Aa*/*Td* /*Pi*	126	12		
*Pg* present + high *Aa*/*Td* /*Pi*	5	182	91.3	97.3

Abbreviations: *Aa* = *A*. *actinomycetemcomitans*, *Pg* = *P*. *gingivalis*, *Td* = *T*. *denticola*, *Pi* = *P*. *intermedia*, NPV = negative predictive value, and PPV = positive predictive value.

^a^ See definitions for “low” and “high” bacterial levels in the footnote of Table 5.

## Discussion

To our knowledge, this investigation is by far the largest epidemiological study employing real-time PCR to study periodontal pathogens in subgingival plaque. The results showed that all species were frequently detectable (>60%) in both no/mild and severe periodontitis groups, with the exception of *A*. *actinomycetemcomitans*. This latter species was harbored in 26% of subjects with no/mild periodontitis, and in 50% of subjects with severe disease. The microorganisms that had the highest bacterial load were the black-pigmented bacteria, whereas the lowest bacterial load was found for *A*. *actinomycetemcomitans*.

In line with our results, previous studies have demonstrated that *P*. *gingivalis*, *T*. *forsythia*, *T*. *denticola*, and *P*. *intermedia* were commonly found in Asian adult populations [3,17,21]. In contrast, wide variations in the prevalence of *A*. *actinomycetemcomitans* have been reported. A low-to-moderate prevalence (10–50%) of this species was observed in the present study, as well as in Japanese [17], eastern Chinese [22], and northeastern Thai populations [5], as opposed to the high prevalence (>80%) observed in northern Chinese [21] and southern Thai populations [3]. These discrepancies could be explained by the differences in the numbers of sampling sites [23], microbial detection methods [11], and geographic locations and/or ethnicity of the study populations [24]. Despite the variations in bacterial prevalence, our study and others consistently observed that *A*. *actinomycetemcomitans* was present at a relatively low level compared to other pathogenic species [3,17,22].

*T*. *forsythia* has been implicated as one of the major etiologic agents of periodontitis [2,3,7,16,21,25]. The present study, consistent with our previous report [14], did not find a significant association between this species and severe periodontitis. It should be noted that when we included only *T*. *forsythia* in the regression model, it was significantly associated with disease in a dose-dependent manner. This is not surprising since *T*. *forsythia* has been known to be strongly correlated with other members of the red complex, *P*. *gingivalis* and *T*. *denticola*, both in terms of bacterial prevalence and quantity [1,6,7]. When the levels of these two species were controlled for, the effect of *T*. *forsythia* was no longer significant. Similarly, a previous study in Finnish also reported no significant association between the prevalence or levels of *T*. *forsythia* and periodontitis after adjusting for the effects of other species [4].

It has been proposed that the amount of pathogenic bacteria must exceed a critical threshold before they can cause disease [10,12]. Our study showed that individuals with the levels of *A*. *actinomycetemcomitans*, *T*. *denticola*, or *P*. *intermedia* below the thresholds were not associated with severe periodontitis any more frequently than those without these microorganisms. In contrast, individuals harboring these species at or above the thresholds experienced a significantly increased likelihood of having disease. We also found that the thresholds for *T*. *denticola* and *P*. *intermedia* were >100 folds higher than the threshold for *A*. *actinomycetemcomitans*, suggesting that this latter species may be more virulent. The relatively low threshold for *A*. *actinomycetemcomitans* was also reported in other studies using different bacterial detection methods, including culture and checkerboard DNA-DNA hybridization [3,25]. Taken together, our findings suggest that for certain species, the levels above the critical thresholds may serve as a better predictor of periodontitis than their presence/absence.

Whereas the threshold effect was observed for *A*. *actinomycetemcomitans*, *T*. *denticola*, and *P*. *intermedia*, the mere presence of *P*. *gingivalis* was associated with severe periodontitis in our study. A currently evolving hypothesis suggests that periodontitis is initiated by a disruption of host-microbe homeostasis [26]. The conversion from homeostasis to a dysbiotic state requires the presence of certain species, called “keystone pathogens”. According to this hypothesis, the keystone species, at very low colonization levels, can modulate host response in ways that alter the amount and composition of subgingival microbiota, thereby triggering periodontal destruction [27]. Our findings coincide with the role of *P*. *gingivalis* as a keystone pathogen. We observed that the presence of *P*. *gingivalis*, even in low amount (less than the 20^th^ percentile of bacterial cells), increased the odds of having periodontitis by 3.2 folds. To increase ORs to a similar level, the amount of *A*. *actinomycetemcomitans* had to be greater than the 66^th^ percentile, while the amount of *T*. *denticola* and *P*. *intermedia* had to reach the 60^th^ and 40^th^ percentiles, respectively. Another interesting finding was that 34 of 214 individuals (16%) developed severe periodontitis in the absence of *P*. *gingivalis*. A subset analysis of these 34 individuals revealed that 22 subjects (65%) were highly colonized by *A*. *actinomycetemcomitans*, *T*. *denticola*, or *P*. *intermedia*, or their combinations, whereas the remaining 12 subjects (35%) harbored low amount of all three species (data not shown). It is possible that under certain circumstances, other members of pathogenic species may take over the role of *P*. *gingivalis* as a keystone pathogen.

Although the keystone pathogens are one of the core requirements for disease initiation, the increased levels of pathogenic species within dysbiotic communities are thought to exhibit synergistic virulence that leads to destructive inflammatory response [26]. Using logistic regression analysis, we demonstrated that in addition to *P*. *gingivalis*, high colonization by *A*. *actinomycetemcomitans*, *T*. *denticola*, and *P*. *intermedia* were significantly associated with severe periodontitis. We also demonstrate for the first time that the levels of *T*. *denticola* and *P*. *intermedia* were strongly correlated in subjects with disease, and that co-infection with these species significantly increased the odds of having severe periodontitis. Compared to subjects with low levels of both species, subjects who were highly colonized by either species alone were 2.5 folds more likely to have disease. The combined effect of being highly colonized by both species further increased the likelihood of having disease to 14.8 folds, indicating a possible inter-species interaction.

Little is known regarding the interaction between *T*. *denticola* and *P*. *intermedia* in periodontitis. A study using fluorescent in situ hybridization has demonstrated that *P*. *intermedia* is found in micro-colonies in the top layer of subgingival plaque, whereas *Treponemes* is located outside the top layer [28]. Their close proximity can be indicative of cell-to-cell adherence or a metabolic synergy. In addition, numerous genes related to motility, metabolism, transport, and outer membrane proteins have shown to be differentially regulated in *T*. *denticola* in the presence of *P*. *intermedia* [29]. Furthermore, dentilisin produced by *T*. *denticola* can cleave the complement factor C3 and the negative complement regulatory protein factor H [30,31], whereas interpain A from *P*. *intermedia* is able to degrade immunoglobulin G and C3 [32,33]. It is plausible that their combined proteolytic activities may be more effective in modulating host immune response than individually. Nevertheless, one should keep in mind that bacterial interactions within subgingival biofilm are complex, and most species are in one way or another correlated to each other [34]. Epidemiological evidence is just a piece of the puzzle to help us comprehend these complex interactions. Further metabolic and functional studies using co-culture or animal models are needed to confirm our findings.

Our study, consistent with previous studies [6,7,34], observed strong correlations between the levels of pathogenic species, indicating that simultaneous detection of these species is more accurate in presenting the risk of periodontitis than are individual species. Using multivariate regression analyses, we demonstrated that a consortium composed of the presence of *P*. *gingivalis* and high colonization by *A*. *actinomycetemcomitans*, *T*. *denticola*, and *P*. *intermedia* was associated with severe periodontitis in this study population. In subjects who are positive for *P*. *gingivalis*, and are highly colonized by *A*. *actinomycetemcomitans*, *T*. *denticola*, and *P*. *intermedia*, the probability of having severe periodontitis is 97%. On the other hand, if persons are negative for *P*. *gingivalis*, and harbor low abundance of *A*. *actinomycetemcomitans*, *T*. *denticola*, and *P*. *intermedia*, they are 91% likely to be periodontally healthy. When individual species were considered, the positive predictive values ranged from 74% to 90%, whereas the negative predictive values were much lower, ranging from 42% to 84%.

Although periodontitis is primarily caused by subgingival plaque bacteria, the disease susceptibility in each individual is influenced by several factors, including smoking and diabetes [20]. Moreover, the varying number of remaining teeth could affect the measurable disease level and the amount of subgingival plaque sampled from each subject. In our study, these confounding factors were controlled for when examining the associations between target species and periodontitis. Additional strengths of this study were the use of real-time PCR for quantitative analysis of target species, and our large sample size, which provided sufficient statistical power to study inter-species interaction. Nevertheless, limitations of the present study are acknowledged. Although this cross-sectional study can demonstrate association, it does not allow any assessment of causal inference. Subsequent longitudinal studies are needed. In addition, the use of pooled plaque samples may have resulted in dilution of the disease-associated bacteria because only a subset of sampling sites was affected by periodontitis. As a result, the association between pathogens and periodontitis might be even stronger than our data indicated.

In conclusion, our findings challenge the role of the red complex as a major etiologic agent of periodontitis. We demonstrated that the presence of *P*. *gingivalis* and high colonization by *A*. *actinomycetemcomitans*, *T*. *denticola*, and *P*. *intermedia* were strong risk indicators of severe periodontitis in our study population. Therefore, the prevention and treatment of periodontal disease in this population should aim at eliminating *P*. *gingivalis* and reducing the levels of the other three species to below the thresholds. Nevertheless, only a small number of species of a much larger microbial consortium was evaluated in our study. Further studies of other putative pathogens and their interactions are required for a full understanding of this polymicrobial disease.
